# Folding principles of genomes

**DOI:** 10.1186/1756-8935-6-S1-O40

**Published:** 2013-03-18

**Authors:** Job Dekker

**Affiliations:** 1 Program in Systems Biology, Department of Biochemistry and Molecular Pharmacology University of Massachusetts Medical School, 364 Plantation Street, Worcester, MA, USA

## 

My laboratory studies how chromosomes are organized in three dimensions. The three-dimensional organization of the genome is critical for regulating gene expression by bringing genes in close spatial proximity to distal regulatory elements such as enhancers. We have developed powerful molecular approaches, based on our Chromosome Conformation Capture technology, to determine the folding of genomes at unprecedented resolution (Kb) and scale (genome-wide).

We have applied these methods to determine the spatial folding of 1% of the human genome (the ENCODE pilot regions) across a panel of cell lines. We discovered that chromosomes fold into extensive long-range interaction networks in which genes are interacting with distal gene regulatory elements. These results start to place genes and regulatory elements, that are often separated by large genomic distances, in three-dimensional context to reveal their functional relationships.

Our analysis of chromosome folding also revealed that chromosomes are compartmentalized in a series of “Topological Association Domains” (TADs) that are hundreds of Kb in size. Loci located within a TAD mingle freely, but interact far less frequently with loci located outside their TAD. TADs appear involved in gene expression, as we found that genes located within the same TAD tend to be co-expressed, but the mechanism(s) by which these domains affect gene regulation is still unknown. TADs represent novel universal and genetically encoded building blocks of chromosomes.

